# Ecological Stoichiometry and Stock Distribution of C, N, and P in Three Forest Types in a Karst Region of China

**DOI:** 10.3390/plants12132503

**Published:** 2023-06-30

**Authors:** Wancai Wang, Yuanying Peng, Yazhen Chen, Shilong Lei, Xiaojun Wang, Taimoor Hassan Farooq, Xiaocui Liang, Chao Zhang, Wende Yan, Xiaoyong Chen

**Affiliations:** 1 State Key Laboratory of Soil Erosion and Dryland Farming on the Loess Plateau, Northwest A&F University, Xianyang 712100, China; 2 College of Arts and Sciences, Lewis University, Romeoville, IL 60446, USA; 3 College of Life Science and Technology, Central South University of Forestry and Technology, Changsha 410004, Chinat.farooq@bangor.ac.uk (T.H.F.);; 4 National Engineering Laboratory for Applied Forest Ecological Technology in Southern China, Changsha 410004, China; 5 Institute of Soil and Water Conservation, Chinese Academy of Sciences and Ministry of Water Resources, Xianyang 712100, China; 6 College of Arts and Sciences, Governors State University, University Park, IL 60484, USA

**Keywords:** nutrient, stoichiometry, Masson pine, Slash pine, karst region

## Abstract

Ecological stoichiometry plays important roles in understanding the nutrient constraints on tree growth and development, as well in maintaining ecosystem services in forests, yet the characteristics of carbon:nitrogen:phosphorous (C:N:P) stoichiometry in forests under karst environment have not been sufficiently evaluated. In this study, concentration, distribution, stocks of Nitrogen (N) and Phosphorous (P), and ecological stoichiometry were studied in three common forest types: Masson pine natural forests (MPNF), Masson pine plantation forests (MPPF), and Slash pine plantation forests (SPPF) in a karst region of southwestern China. Results showed that N concentrations were higher in overstory than in understory and litter in the studied forests. However, P concentration was relatively low in overstory component of the forested ecosystems. Meanwhile, the N and P concentrations were higher in SPPF in the stem and litter, while these contents were higher in MPPF and MPNP in the overstory and understory. The N and P stocks ranged from 5.7–6.2 t ha^−1^, and 0.5–0.6 t ha^−1^ in the examined forests. The ecological stoichiometry of C:N:P in the three forest types was similar in litter (46–49:2:1), and relatively steady in soil (250–320:13–16:1) and tree leaf (100–200:14–20:1). Soil P status was the primary limiting factor in affecting tree growth in MPPF and SPPF (N:P ratio > 16), while both N and P conditions were the main restrictive factors in MPNP (N:P ratio = 15) in the study area. Our study provides scientific references and useful datasets of C:N:P stoichiometry for sustainable management of forest ecosystems in karst regions.

## 1. Introduction

Ecological stoichiometry, dealing with the mass balance of energy and nutrient elements in organisms and their interaction in nature [1], provides important and new insights to regulate organism development and biogeochemical cycle in terrestrial ecosystems [2,3]. The nutrient cycle through various compartments in forest ecosystems is a major determinant of the ecosystem structure and function. The relationship between carbon (C), nitrogen (N), and phosphorus (P) is particularly important because they are essential structural elements in all organisms, and ecosystem dynamics are frequently limited by these three elements [4,5]. Furthermore, C:N:P stoichiometry can significantly influence many important ecosystem processes such as mineralization, litter decomposition, and plant community succession [6,7]. Therefore, ecological stoichiometry of C, N, and P has been widely employed as a useful indicator to describe nutrient status and cycle, soil enzyme activity, and soil health in forest ecosystems [8].

The variation of C:N:P stoichiometry in forest ecosystems could, in turn, affect the ecosystems’ structure and biogeochemical cycles [9]. The changes of C:N ratio in litter might regulate the terrestrial C cycle through the alteration on decomposition processes [10]. Decreasing the C:N ratio in plant litter or mineral soils may be conducive to microbial decomposition. Conversely, microbial decomposition may be inhibited by increasing the C:N ratio in plant litter or mineral soil [11]. Increased N:P ratios in leaf, litter, or soil organic matter could lead to a shift in ecosystem nutrient limitation from N towards P, ultimately resulting in changes in composition, structure, and function of the ecosystems [12,13]. The vegetation types had significant influences on soil nutrient states and ecological stoichiometry [14]. Zeng et al. [15] reported that the accumulation of soil organic matters in cropping communities significantly varied among vegetation types because of differences in nutrient contents in plant organs and thereafter decomposition processes of aboveground and belowground litter debris. Consequently, the identification of stoichiometric structure and variation of C, N, and P in a forest ecosystem could provide better understanding of interactions and dynamics of nutrients in the ecosystem under a changing environment [6,10,16].

Karst regions cover ~12% of the global land area and are characterized by rugged landscape, poor soil, and sparse vegetation community [17]. Since 1990, ecological degradation in karst areas has rapidly occurred worldwide due to increase of human population and heavy anthropogenic disturbances [18,19]. It is a particular case in karst regions of Southwest China where the vegetation communities have been seriously damaged, and the water and soil erosion have led to a large loss of nutrients from the soils [20]. Although some studies have been conducted to examine the effect of land use changes and restoration practices on nutrients cycling and soil fertility in karst regions, the quantitative distribution and relationship of C:N:P stoichiometry in different forest types under karst environments are still less documented [21,22,23].

In the current study, a field investigation was conducted in a karst region in Southwestern China, focusing on the C:N:P stoichiometry of three common pine forest types in this region: Masson pine natural forest (MPNF), Masson pine plantation forest (MPPF), and Slash pine plantation forest (SPPF). We hypothesized that the amount, distribution, and stoichiometry of N and P varied among different forest types due to differences in genetic and biological characteristics. The purpose of this study was to examine the variations of ecological stoichiometry of C, N, and P in the selected forest ecosystems. Our specific objectives were: (1) to quantify the concentration, distribution, and stocks of N and P in various organs and components of the studied forests; and (2) to explore the differences of stoichiometry of C, N, and P among the three forest ecosystems. The results of this study could provide scientific reference for further understanding of nutrient biogeochemistry and sustainable management of forest ecosystems in this area.

## 2. Materials and Methods

### 2.1. Study Site

The study was carried out at the Longli Forest Farm, Longli County, Guizhou Province, China (26°22′–26°45′ N, 106°45′–107°11′ E). The Farm covers a total of 13.3 thousand hectare of forestlands. The elevation of the region ranged from 770 to 1775 m. The climate of the study site is characterized by a warm winter and a cool summer. The average annual temperature is 14.8 °C, with January and July averaging 4.8 °C and 23.5 °C, respectively. The average annual rainfall is 1089.3 mm. The soil is classified as a cinnamon clay loam, developed on limestone, and has an average depth of more than 1 m with pH value of 5–6 on the topsoil (0–15 cm).

Since 1995, afforestation activities have been progressing, and a large number of forest plantations have been established on the Farm. In this study, three common types of pine forest (SPPF, MPPF, and MPNF) have been selected, and they were about 20 years old. The SPPF and MPPF were planted as pure plantations with initial stand densities of 2 m × 3 m and 3 m × 3 m, respectively. The MPNF were naturally regenerated stands from a harvesting site, with 90% of tree species being Masson pine. The three selected forest types were about 500 m apart. The main shrub species in these forests were *Smilax china* L., *Rhododendron simsii* Planch., *Quercus fabri* Hance, and *Lyonia ovalifolia* (Wall.) Drude. The main herb species were *Imperata cylindrica* (L.) P. Beauv., *Woodwardia japonica* (L.f.) Sm., and *Dicranopteris linearis* (Burm.) Underw. The information on the characteristics of the sites and the characteristics of the stands of the three forest types is presented in Table 1.

### 2.2. Experimental Design and Sampling Collection

The current study was executed with a completely randomized design. Three 20 m × 20 m replicate plots were set up for each of the examined forest types (SPPF, MPPF, and MPNF) in the study site. To minimize the variability between the plots, all of the forest stands were characterized by similar stand age, slope, elevation, soil type, soil texture, and topography. These forest plots were at least 500 m away from each other and used for the subsequent plant and soil sampling. Three 2 m × 2 m subplots were randomly established within each plot of a forest type for collecting shrub samples. Three 1 m × 1 m quadrats were randomly established for each plot to take herbaceous samples. To collect litter samples on the forest floor, five 1 m × 1 m quadrats were randomly set up in each plot of a forest type. 

A soil auger (5 cm diameter) was used to collect soil samples from three locations in each plot of an examined forest type. The locations for sampling soils were about 2 m apart from each other. Therefore, a total of 3 replicate stand plots, 9 subplots, 9 quadrats, and 15 quadrats were established to collect plant samples in the overstory, shrub, herb, and litter layers in each forest type, respectively, in this study.

All plant samples were taken by using a harvesting method when forest biomass was measured [23]. Briefly, the diameter at breast height (DBH ≥ 5 cm, 1.3 m) of all trees in each plot was measured, and the trees were also classified and counted by species. Six sample trees representing the DBH distribution in the plot were cut down and the roots of each sampled tree were carefully excavated out. The overstory tree samples were collected from these cut down sampling trees and divided into leaf, branch, stem, and root organs. The detailed sampling process can be found in the reference [23]. The understory (small tree, shrub, and herbaceous) plant samples were mixed and divided into two parts: aboveground component and belowground component. Litter samples were not further classified into leaf and twig components, and they were collected as a whole mixed part. Soil samples were taken from different mineral soil layers (0–15, 15–30, 30–45, and 45–60 cm depth) using a soil auger (5 cm in diameter), separately. The soil samples taken from the same depth in three locations within a plot were pooled and formed as one mixed soil sample. 

### 2.3. Chemical Analysis

In the laboratory, all plant samples were dried in an oven at 65 °C, grounded, and passed through a 0.5 mm mesh screen. Soil samples were air-dried at room temperature (25–28 °C), crushed, and passed through a 2 mm sieve. The C concentration in plant and soil samples was determined by a modified dichromate oxidation method [24]. The N concentration was determined by Kjeldahl acid digestion method after extraction with sulfuric acid on a distillation unit [25]. The P concentration was determined colorimetrically using a UV-visible spectrophotometer (model UV-2300, Techcomp Com, Shanghai, China) after the digestion with H_2_SO_4_ and HClO_4_ as described by Parkinson & Allen [26].

### 2.4. Data Analysis

The C data came from our previous study [23]. Analysis of variance (ANOVA) and multiple comparison (LSD test) were used to statistically test the effects of different forest types on the N and P concentrations of plant and soil depth, as well as the ratios of C:N, C:P, and N:P. All sample data was transformed when needed to meet assumptions of normality and homogeneity. Logarithmic transformations were performed on the original N and P data to meet the normality and isotopic assumptions of ANOVA. All differences were considered significant at *p* < 0.05. Statistical analysis was performed using the SAS Statistics Package (SAS Institute, Inc., Cary, NC, USA 1999–2001) and SPSS 20. 

## 3. Results

### 3.1. The Concentration, Distribution, and Stocks of N and P

The concentrations of N were significantly lower in leaf and higher in stem in SPPF than those of MPNF and MPPF, respectively (*p* < 0.05). No statistically significant difference of N concentration was found in branch among the studied forests (*p* = 0.1424). The concentration of N in roots significantly differed among the forest types, with a decrease order of MPNF > SPPF > MPPF. The SPPF had significant lower concentration of P in leaf and branch than those of MPNF and MPPF. The P concentration was significantly higher in root of MPNF than of SPPF and MPPF. There was no significant difference of P concentration in tree stems among the three types of forests (*p* = 0.1881) (Table 2). No significant differences of N and P concentrations were found in shrub, herb, and litter components among the three forests (*p* > 0.05), except in SPPF where P concentration in belowground components of shrub was significantly lower than that of MPNF and MPPF (*p* = 0.0064) (Table 2). Additionally, the concentrations of N and P in soils were higher in SPPF than in MPNF and MPPF. 

**Figure 1:**
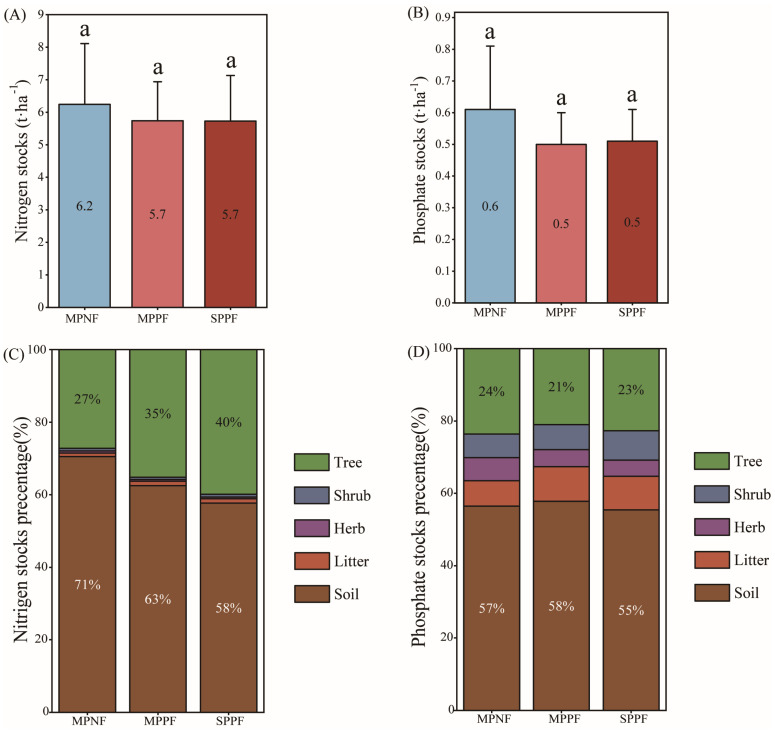
The variation of nitrogen (N) and phosphorous (P) stocks (**A**,**B**) and their percentage (%) in various ecosystem components (**C**,**D**) in the three forest types in the study site. Different lowercase letters represent the significant difference in the three forest types. The vertical bars are the standard errors of the mean.

The stocks of N and P ranged from 5.7–6.2 t ha^−1^, and 0.5–0.6 t ha^−1^ in the three examined forests (Figure 1A,B). The MPNF stored a relatively high percentage of N in soil, while the MPPF and SPPF stored a high percentage of N in tree (Figure 1C,D). The stocks of N and P were similar in various tree organs (Table 3 and Table 4). 

### 3.2. The Stoichiometric Characteristics of C, N, and P

**Figure 2:**
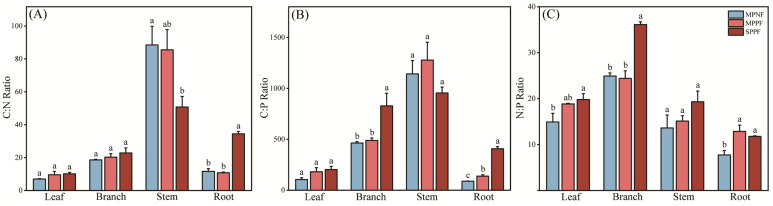
The variation of C:N, C:P, and N:P ratio (**A**–**C**) in various tree organs in the three forest types. Different lowercase letters represent the significant difference at the three forest types. The vertical bars are the standard errors of the mean.

Overall, the C:N ratio was significantly different in root (*p* < 0.0001), marginally significantly different in stem (*p* = 0.047), but not different in leaf (*p* = 0.2016) and branch (*p* = 0.4354) in the studied forests (Figure 2A). Significant differences of the C:P and N:P ratios were found in branch (*p* = 0.008) and root (*p* = 0.0215), marginally in leaf (*p* = 0.08), but not in stem (*p* = 0.2519) (Figure 2B). The C:N ratio of stem was significantly higher in MPNF and MPPF than in SPPF, but C:P ratio in leaf and branch were lower in MPNF and MPPF than in SPPF. For root, the C:N and C:P ratios were significantly larger in SPPF than in other examined forest types. The SPPF had a significantly higher N:P ratio in all tree organs than those for MPNF and MPPF, expect in root where MPPF had the higher N:P ratio compared to that of SPPF (Figure 2C).

**Figure 3:**
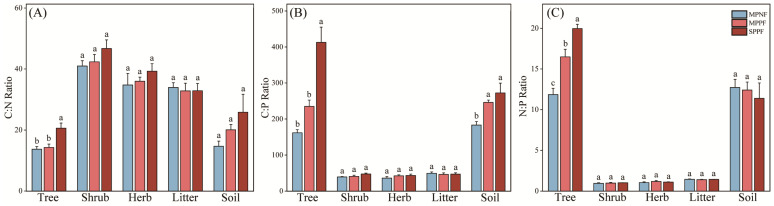
The variation of C:N, C:P, and N:P ratio (**A**–**C**) in different components of the three forest types. Different lowercase letters represent the significant difference at the three forest types. The vertical bars are the standard errors of the mean.

There were no significant differences of C:N, C:P, and N:P ratios (*p* > 0.05) in shrub, herb, and litter layers in the three forest types (Figure 3). Significantly statistical differences of C:N, C:P, and N:P ratios were found in tree among the three forest types (*p* < 0.05) (Figure 3), with a decreasing order of SPPF > MPPF > MPNF. Significantly statistical differences of C:P ratios were found in soil among the three forest types (*p* < 0.05), with a decreasing order of SPPF > MPPF > MPNF (Figure 3B). The C:N ratio ranged from 14–25 in soil of the three forest types and was higher in the root organ and tree component of the SPPF. 

The ecological stoichiometry of C, N, and P elements in litter was similar in the three forest types with a C:N:P ratio of about 47:2:1 (Table 5). The patterns of C:N:P ratios in leaf and soil were relatively steady in the three examined forests with a decreasing order of SPPF > MPPF > MPNF. 

## 4. Discussions

The highest N and P concentrations were found in leaf in various tree organs all three examined forest types while stem had the lowest N and P contents in the current study. The results were consistent with the findings in previous studies [27,28]. It is because the leaf is an active organ for metabolic processes such as photosynthesis, cellular respiration, and transpiration, which required large quantities of nutrients to sustain these activities. However, the stem is a storage organ with the deposition of cellulose, hemicellulose, lignin, and pentosanes [29]. We also found the concentration of N elements was higher in overstory than in understory and litter layers, while P concentration was significantly lower in the overstory than in both understory and litter layers [30]. Consequently, higher C:P and N:P ratios were expected in the overstory than in the understory and litter layers. Such stoichiometric features were likely related to the different composition of these plant components in a forest. Usually, the overstory in a forest has a high proportion of stem and a low proportion of leaf when compared to the understory and litter, which have relatively high percentage of leaf and low proportion of woody stem [23,31]. Han et al. [32] and Elser et al. [6] reported that leaf P concentration declined with increasing plant size as small plants maintained higher nutrient concentrations than large trees.

N and P levels varied among leaves, stems, and roots because of the structural and physiological differences of these tree organs. The levels of these elements also changed as a function of annual mobilization from storage to growing sites within the plant body [33]. The leaf N:P ratio has often been used to represent nutrient limitation during plant growth [34]. Leaf N:P ratios were negatively related to relative growth rate in different forests [6,28]. Because of fast growth, the P concentrations in Eucalyptus leaves were reduced with tree growth, leading to an increase of N:P ratios in leaf [28]. A similar result was found in a study with tree species of Betula pendula and Pinus contorta [35].

In addition, the C:N ratio ranging from 14–25 in soil of the three forest types was consistent with previous studies where the C:N ratio is intermediate between 16 and 44 at the forest soil. The result showed that the C:N ratio was higher in the root organ and tree component of the SPPF. This is because the Slash pine is the fast-growing tree, and the root was the main organ of nutrient absorption and utilization, leading to an increase of C:N ratio in root. Leaf N:P ratio has been used as an important indicator to distinguish if the forests were in N-limitation or P-limitation status. Terrestrial plant leaves tended to have an optimum N:P ratio of 14–16 by mass [36]. If N:P ratios < 14, the plants were suggested to be in N-limitation; N:P ratios > 16 suggests P-limitation; and N:P ratios between 14 and 16 suggest both N- and P-limitation [28,37]. In the present study, the average leaf N:P ratios were 15, 19, and 20 in MPNF, MPPF, and SPPF stands (Figure 2), respectively. These results suggested P element was the primary limiting factor in affecting tree growth in MPPF and SPPF stands. Both N and P elements were the limiting factors in MPNF stands as the leaf N:P ratio was 15. Our results were in line with the findings in reported by Zhang et al. [38], who reported that N element was the limiting factor in the grasslands, P in the forests, and both N and P in the shrub lands in this region.

It is well known that plant nutrient status is mostly limited by N and P availability in soil [39]. For example, the dynamics of plant C, N, and P were primarily influenced by soil N and P supply, especially in tropical forests [40] and subtropical regions [28]. Our study results show the SPPF had the low N and P concentrations in leaf component of overstory tree, and relatively high N and P concentrations in soil among the three forest types, indicating a negative relationship in N and P nutrients between above ground and soil in this forest stand. These results might be attributed to the differences between growth rate and nutrients availability in soil in SPPF stands. Fast growth of above parts of plants often resulted in a reduction of element concentration in leaves because of a dilution effect, meaning the accumulation rate of nutrient elements was less than the accumulation rate of biomass production in leaf organ. Thus, the element concentration in leaves per mass basis gradually declined with tree growth. On the other hand, the fast growth of plants led to more activity and metabolic processes underground, which resulted in an increase of nutrient availability in soils. In addition, the retranslocation of nutrients between soil and plant confirmed these relationships. Fan et al. [28] found the similar retranslocation patterns for N and P contents in the leaves of different tree species.

A high N:P ratio was found in overstory, but low N:P ratio was observed in understory and litter components in the studied site. Specifically, the N:P ratios were 20–28 in overstory, and about 1 in understory and litter layers in the three forest types (Figure 3). These results confirmed that serious P deficiency occurred in these forests (N:P ratios > 16 suggests P-limitation, mentioned above) and P element was the primary limiting factor in affecting tree growth and forest biomass productivity in the karst region. However, the low N:P ratios in the understory and litter components of the forest ecosystems suggest this component might be an important P resource for maintaining the P nutrient level in this site. Therefore, how to appropriately manage the understory and litter layer in order to maintain P fertility level in the forestlands should be a critical target for the local farmers and governments to sustainably manage the forest resources in the karst area.

## 5. Conclusions

In summary, the concentration, stock, and stoichiometric ratio of N and P varied among different tree organs and forest components in the three forest types. suggesting the N and P stoichiometry may be inherently or biologically flexible in forest ecosystems in this region. Because of relative stability of C content in tree organs, the relatively high N and P in leaf but low contents in stem resulted in low C:N, C:P, and N:P ratios in leaf, and high C:N and C:P ratios in stem in the examined forests. The C:N:P ratio was quite similar in litter (46–49:2:1), and relatively steady in soil (250–320:13–16:1) and tree leaf (100–200:14–20:1) in the three forest types. MPNF has more advantages in terms of P nutrient stock and N:P ratio than do MPPF and SPPF, suggesting the MPNF could be a suitable forest type for restoring and conserving soil nutrients in the study area. The relatively high N:P ratios in leaf and soil (N:P ratio > 16) indicated that P element was the primary limiting factor in affecting forest growth, suggesting nutrient stoichiometry might be particularly influenced by P-limitation in the study forests. Our results provide a scientific basis and datasets of ecological stoichiometry for sustainable management of forest ecosystems in karst regions.

## Figures and Tables

**Table 1:** Stand characteristics of the three forest types in the study site.

Forest Types	Age (Year)	Tree Density (Tree/ha)	DBH (cm)	Tree Height (m)	Shrubs	Herbs
MPNF	18	2242 (125.83)	13.0 (1.09)	12.2 (0.63)	1,2,3,4,5	9,10,11,12
MPPF	19	1217 (625.17)	17.5 (0.35)	15.6 (0.59)	1,2,3,4,6	9,10,11,12
SPPF	20	950 (163.94)	21.0 (1.13)	14.8 (0.51)	2,4,5,7,8	9,10,11

Notes: Mean (standard error). Shrubs and herbs species: 1, *Smilax china* Linn. 2, *Rhododendron simsii* Planch. 3, *Quercus fabri* Hance. 4, *Lyonia ovalifolia* (Wall.) Drude var. elliptica. 5, *Styrax japonicus* Siebold & Zucc. 6, *Castanea seguinii* Dode. 7, *Albizzia corniculata* (Lour.) Druce. 8, *Gaultheria trichophylla* Royle. 9, *Imperata cylindrica* (L.) P. Beauv. 10, *Woodwardia japonica* (L. f.) Sm. 11, *Dicranopteris linearis* (Burm.f.) Underw. 12, *Dryopteris Adanson*.

**Table 2:** The concentration, distribution of Nitrogen (N), and Phosphorous (P) in various tree organs and ecosystem components in the three forest types in the study site (g kg^−1^).

Element	Forest Types	Overstory		Stem	Root	Understory		Litter	Soil			
		Leaf	Branch			Shrub	Herb		0–15 cm	15–30 cm	30–45 cm	45–60 cm
N	MPNF	60.79a (1.8)	22.98a (0.2)	5.21a (0.6)	42.03a (0.6)	8.80a (0.5)	9.84a (0.4)	10.70a (0.2)	0.50a (0.1)	0.42a (0.1)	0.33a (0.1)	0.29a (0.1)
	MPPF	67.34a (0.5)	23.59a (0.6)	5.12a (0.1)	5.36b (1.9)	9.39a (0.8)	9.28a (1.0)	11.23a (0.2)	0.44a (0.1)	0.32a (0.1)	0.32a (0.1)	0.35a (0.1)
	SPPF	51.13b (0.5)	22.34a (0.1)	10.39b (0.1)	12.54c (0.1)	8.19a (0.1)	8.69a (0.4)	12.01a (0.5)	0.56a (0.2)	0.42a (0.1)	0.42a (0.1)	A/N
P	MPNF	4.22a (0.6)	0.92a (0.0)	0.42a (0.1)	5.60a (0.7)	9.17a (0.2)	9.51a (0.7)	7.39a (0.4)	0.03a (0.0)	0.04a (0.0)	0.03a (0.0)	0.03a (0.0)
	MPPF	3.57a (0.1)	0.97a (0.1)	0.34a (0.0)	4.23b (0.4)	9.65a (0.3)	7.80b (0.3)	8.00a (0.2)	0.03a (0.0)	0.03a (0.0)	0.03a (0.0)	0.03a (0.0)
	SPPF	2.60b (0.1)	0.62b (0.0)	0.55a (0.1)	1.06b (0.0)	8.06b (0.1)	7.83b (0.3)	8.35a (0.4)	0.04a (0.0)	0.04a (0.0)	0.04a (0.0)	A/N

Notes: Mean (standard error). Different letters in the same column indicate significant differences at *p* < 0.05 level for the same element among different forest types.

**Table 3:** Nitrogen stocks (t ha^−1^) in various tree organs and ecosystem components in the three forest types in the study site.

Forest Types	Leaf	Branch	Stem	Root	Tree	Shrub	Herb	Litter	Soil
MPNF	0.43b (0.01)	0.42b (0.01)	0.35b (0.05)	0.50a (0.10)	1.70a (0.50)	0.04a (0.00)	0.04a (0.00)	0.06a (0.00)	4.40a (0.98)
MPPF	0.68a (0.01)	0.85a (0.03)	0.41b (0.07)	0.07a (0.05)	2.02a (1.13)	0.03a (0.00)	0.03a (0.00)	0.07a (0.00)	3.59a (0.75)
SPPF	0.46b (0.01)	0.33b (0.01)	1.31a (0.21)	0.19a (0.10)	2.28a (1.03)	0.04a (0.00)	0.03a (0.00)	0.07a (0.00)	3.31a (0.87)

Notes: Mean (standard error). Different letters in the same column indicate significant differences at *p* < 0.05 level for the same element among different forest types.

**Table 4:** Phosphate stocks (t ha^−1^) in various tree organs and ecosystem components in the three forest types in the study site.

Forest Types	Leaf	Branch	Stem	Root	Tree	Shrub	Herb	Litter	Soil
MPNF	0.03a (0.00)	0.02a (0.00)	0.03a (0.00)	0.07a (0.00)	0.14a (0.01)	0.04a (0.00)	0.04a (0.00)	0.04a (0.00)	0.35a (0.09)
MPPF	0.04a (0.00)	0.04a (0.00)	0.03a (0.00)	0.01a (0.00)	0.11a (0.01)	0.03a (0.00)	0.02a (0.00)	0.05a (0.00)	0.29a (0.08)
SPPF	0.02a (0.00)	0.01a (0.00)	0.07a (0.00)	0.02a (0.00)	0.12a (0.01)	0.04a (0.00)	0.02a (0.00)	0.05a (0.00)	0.28a (0.08)

Notes: Mean (standard error). Different letters in the same column indicate significant differences at *p* < 0.05 level for the same element among different forest types.

**Table 5:** C:N:P ratios in selected leaf organ, litter, and soil components of the examined three forest ecosystems in the study site.

Forest Type	Leaf	Litter	Soil
MPNF	100:14:1	49:2:1	255:16:1
MPPF	181:19:1	46:2:1	310:14:1
SPPF	200:20:1	47:2:1	317:13:1

## Data Availability

The data is contained within the manuscript.
